# Nonautomated Blood Cultures in a Low-Resource Setting: Optimizing the Timing of Blind Subculture

**DOI:** 10.4269/ajtmh.20-0249

**Published:** 2020-11-30

**Authors:** Sien Ombelet, Marjan Peeters, Chhundy Phe, Achilleas Tsoumanis, Chun Kham, Syna Teav, Erika Vlieghe, Thong Phe, Jan Jacobs

**Affiliations:** 1 Department of Clinical Sciences, Institute of Tropical Medicine, Antwerp, Belgium;; 2 Department of Immunology, Microbiology and Transplantation, KULeuven, Leuven, Belgium;; 3 Sihanouk Hospital Center of Hope, Phnom Penh, Cambodia;; 4 Department of General Internal Medicine, Infectious and Tropical Diseases, University Hospital Antwerp, Antwerp, Belgium

## Abstract

Laboratory procedures for blood cultures in a hospital in Phnom Penh were adapted to optimize detection of *Burkholderia pseudomallei*, an important pathogen in this setting. The effects of these changes are analyzed in this study. Blood cultures consisted of two BacT/ALERT bottles (bioMérieux, Marcy-l’Etoile, France). Growth was detected visually by daily inspection of the bottles. In 2016, the aerobic–anaerobic pair (FA/FN FAN) was substituted by an aerobic pair of BacT/ALERT FA Plus bottles. Blind subculture (BS) (subculture in the absence of visual growth) was advanced from day 3 to day 2 of incubation in July 2016. In July 2018, it was further advanced to day 1 of incubation. From July 2016 to October 2019, 9,760 blood cultures were sampled. The proportion of cultures showing pathogen growth decreased from 9.6% to 6.8% after the implementation of the laboratory changes (*P* < 0.001). Advancing the BS from day 3 to day 2 led to an increased proportion of pathogens detected by day 3 (92.8% versus 82.3%; *P* < 0.001); for *B. pseudomallei*, this increase was even more remarkable (92.0% versus 18.2%). Blind subculture on day 1 similarly increased the proportion of pathogens detected by day 2 (82.9% versus 69.0% overall, 66.7% versus 10.0% for *B. pseudomallei*; both *P* < 0.001). However, after implementation of day 1 subculture, a decrease in recovery of *B. pseudomallei* was observed (12.4% of all pathogens versus 4.3%; *P* < 0.001). In conclusion, earlier subculture significantly shortens time to detection and time to actionable results. Some organisms may be missed by performing an early subculture, especially those that grow more slowly.

## INTRODUCTION

Bloodstream infections (BSIs) lead to considerable morbidity and mortality worldwide.^1–3^ Blood cultures are currently the most important tool for diagnosis of BSIs.^4^ Furthermore, they enable performing antibiotic susceptibility testing of the causative pathogens, which is instrumental in patient management and antibiotic stewardship. However, in many low-resource settings, blood culture surveillance is lacking.^5–7^ Automated equipment for blood culture incubation is too expensive and its maintenance too exigent for these settings.

Since 2007, blood cultures are collected in Sihanouk Hospital Center of Hope (SHCH), a nongovernmental organization 30-bed hospital in Phnom Penh, Cambodia. A pair of BacT/ALERT blood culture bottles (bioMérieux) are sampled from separate venipunctures and incubated in a static incubator, as opposed to an automated incubator. The bottles are daily inspected visually to detect changes in the color indicator on the bottles. These procedures were outlined in a previous publication, describing results of the blood culture surveillance for the period 2010–2015 in SHCH.^8^

**Figure 1.**
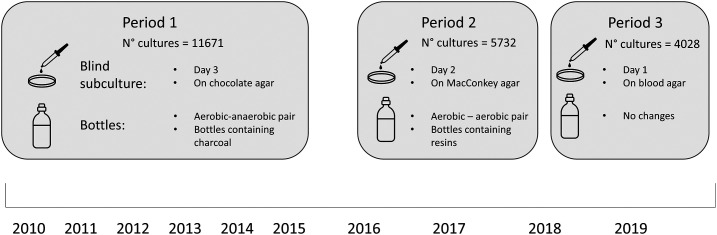
Procedural changes implemented during the three time periods of blood culture surveillance. The detailed results of period 1 are described in a previous publication.^8^ This figure appears in color at www.ajtmh.org.

One of the conclusions of the previous study was that detection of *Burkholderia pseudomallei*, a key pathogen in this setting and the causative agent of melioidosis, is tardive compared with other pathogens, despite results from another study, showing fast growth of *B. pseudomallei* in automated blood culture systems.^9^ Another finding from our surveillance was the increased value of aerobic bottles compared with anaerobic bottles, especially for the detection of non–glucose-fermenting Gram-negative organisms such as *B. pseudomallei*. Some procedural changes were therefore implemented in the laboratory, to increase and speed up detection of this pathogen (Figure 1).^8^

First, instead of performing a blind subculture (BS) (i.e., a subculture of all aerobic bottles in the absence of visual signs of growth) on day 3 of incubation, it was decided to advance the BS to day 2 of incubation. Second, the aerobic–anaerobic pair of bottles was replaced by an aerobic–aerobic pair. Moreover, the BS medium was changed from chocolate agar to McConkey agar because of the high number of contaminants found on chocolate agar. Incidentally, the bottle types used from 2010 to 2015 (the charcoal-containing BacT/ALERT FA FAN and FN FAN) were withdrawn from the market by bioMérieux and replaced by the resin-containing BacT/ALERT FA Plus bottles. These changes were consecutively implemented from April to June 2016.

In this study, we describe how these procedural changes led to differences in time to detection (time between incubation and first sign of growth) and in “time to colonies” (time between incubation and first recovery of colonies on agar) of key pathogens. An interim analysis 2 years after implementation led to additional changes in the procedures (July 2018): BS was further advanced to day 1 of incubation and was performed on blood agar instead of McConkey agar, to demonstrate earlier growth of *Staphylococcus aureus* BSI. Figure 1 summarizes the procedural changes over the different surveillance periods.

## METHODS AND MATERIALS

### Laboratory materials and procedures.

BacT/ALERT FA Plus bottles (reference number 410851) were used for blood culture sampling. For adults, a set of blood culture bottles (2 × 10 mL in two FA Plus bottles) was sampled. For children 15 years of age or younger, guidelines recommend sampling of only one (aerobic) bottle. However, for 66% of cultures in children in SHCH (*n* = 128), two bottles had been sampled. On arrival in the laboratory, bottles were weighed and incubated in a static incubator at 35°C ± 1°C for 7 days. Bottles were inspected twice daily for signs of growth (such as color change of the indicator). Bottles with signs of growth were further processed by performing Gram stain of the bottle and subculture on solid media appropriate according to the Gram stain result. A BS was performed on McConkey agar for each bottle on day 2 of incubation from July 2016 to July 2018. From July 2018 to November 2019, BS was performed on blood agar for each bottle on day 1 of incubation.

### Laboratory methods for identification and antibiotic susceptibility testing.

After growth of colonies on subculture, bacterial isolates were identified using conventional microbiological techniques and Analytical Profile Index (API^®^) tests (bioMérieux) when necessary.^10^ Antibiotic susceptibility was tested with disk diffusion. Antibiotic disks used were Neo-Sensitabs™ (Rosco Diagnostica, Taastrup, Denmark).

### Inclusion and exclusion criteria.

Indications for blood culture sampling were based on the systemic inflammatory response syndrome criteria: presence of tachycardia, tachypnea, fever, or hypothermia (temperature > 38°C or < 36°C); altered mental status; and presence of inflammatory parameters such as leukocytosis or leukopenia.^11^ Homemade glass bottles and solitary bottles from adults (or children aged > 15 years) were excluded as they do not reflect optimal blood culture methods and to facilitate comparison with the period of 2010–2015, when these bottles were also excluded from the analysis.^8^ Cultures are sampled free of charge, excluding any economical bias to the patient population included in the study.

### Data collection and extraction.

Patient demographic data, clinical information (such as use of antibiotics 14 days before blood culture sampling) as well as detailed microbiological data were extracted from the laboratory information system (structured query language [SQL]) into Microsoft^®^ Excel^®^ for Office 365 (Microsoft Corp., Redmond, WA) for the period July 2016 up to November 2019. Doubtful results and likely errors were verified with the laboratory notebooks. Only paired bottles were considered for analysis, except for children younger than 15 years of age, for whom also single bottles were included. Homemade bottles were excluded from analysis.

### Blood volume measurement and calculation.

Blood culture bottles were measured on arrival in the laboratory. The weight was noted in the laboratory logbooks and entered in the SQL database (Oracle Corporation, Redwood City, CA). The volume of blood was calculated by subtracting the mean empty weight of a BacT/ALERT FA Plus bottle (measured mean weight = 61.9 g, SD ± 0.12 g) and next dividing the result by the density of blood (= 1.06 g mL^−1^).^12^ Bottles from children aged ≤ 15 years were not included in the analysis of the blood volume, as guidelines for optimal volume in children differ.^13^

### Incubation delay.

Day of blood culture request and day of reception in the laboratory were compared to assess possible delay in incubation (needle-to-incubator time).

### Statistical analysis.

Export of the data from SQL to Microsoft Excel was cleaned and imported into *R* (R Foundation for Statistical Computing, Vienna, Austria). Statistical tests were performed using *R* version 3.6.1 (July 5, 2019). Univariate differences in proportions, means, and median values were assessed using chi-square test, Student’s *t*-test, and Wilcoxon rank-sum test, respectively. A segmented logistic regression, modeling the effect of one (July 2016) or two interventions (July 2016 and July 2018), on pathogen growth rate was performed. To assess for the impact of other variables on pathogen growth, such as age, gender, hospitalization status, prior antibiotic use, volume sampled, and delay in incubation, multivariate logistic regression models were calculated for each surveillance period separately and for periods 2 and 3 combined. Presence of a trend over time in the total number of cultures sampled and absolute number of grown pathogens was assessed using Poisson regression. Trends over time in pathogen growth rate and recovery on BS were assessed using logistic regression.

### Definitions.

Definitions of blood culture–related terms used in this study are given in Table 1. For further reference, the surveillance period of 2010–2015 is called “period 1,” the surveillance period of July 2016 to June 2018 is called “period 2,” and the surveillance period of July 2018 to October 2019 is called “Period 3.” The proportion of blood cultures showing growth of a pathogen will be referred to as “pathogen growth rate.”

**Table 1:** Definitions used in this study^8^

Term	Definition
Blood culture	Adults: a collection of blood culture bottles (2 or more) sampled at the same time
	Children aged ≤ 15 years: one blood culture bottle
Blood culture set	Two aerobic bottles sampled from the same patient at the same time
Solitary bottle	One bottle sampled from the same (adult) patient at the same time
Cancelled blood cultures	Blood culture bottles that arrived in the laboratory but were not further worked up because of cancellation by the treating physician
Suspected BSI episode	A suspected BSI episode was defined as all blood cultures sampled within a 14-day interval from the first sample, unless growth (see in the following text)
Culture-confirmed BSI episode	A BSI episode was defined as 1) the initial recovery of a pathogen from a suspected BSI episode, 2) the recovery of a pathogen different from the initial pathogen ≥ 48 hours after the recovery of the initial pathogen, or 3) the recovery of the same pathogen after at least a 14-day interval since the previous grown culture with this pathogen^8^
BS	A subculture performed in the absence of any visual signs of growth (in this case, change in color of the growth indicator)
Contamination rate	Skin and environmental bacteria (coagulase-negative staphylococci, *Corynebacterium* species, and *Bacillus* species) were categorized as blood culture contaminants.^18^ The rate of contaminants was defined as the number of bottles grown with contaminants divided by the total number of bottles collected (as each bottle was sampled by a separate venipuncture) and expressed as a percentage
Pathogen growth rate	The number of blood cultures demonstrating growth of a pathogen, divided by the total number of blood cultures and expressed as a percentage
Period 1	Period of blood culture surveillance from 2010 to 2015; BS of aerobic bottle done on day 3 of incubation
Period 2	Period of blood culture surveillance from July 2016 to June 2018; BS done on day 2 of incubation
Period 3	Period of blood culture surveillance from July 2018 to October 2019; BS done on day 1 of incubation
Day of incubation	Days of incubation were mentioned to indicate for instance the time-to-detection. They were defined as follows:
	Day 0 = reception in the laboratory
	Day 1 = after 1 night of incubation
	Day 2 = after 2 nights of incubation
	Day 3 = ….
Time to detection	The time between reception of the bottle in the laboratory and the moment that growth of a pathogen is detected and can be reported. For bottles showing growth first on BS, the time to detection is the day after the BS, that is, the day colonies are witnessed on BS.
Time to colonies	The time between reception of the culture in the laboratory and the moment that colonies are available on solid medium (agar) for further testing
Community-acquired and healthcare-associated BSI episodes	Community-acquired and healthcare-associated BSI were defined according to the day of sampling, that is, at ≤ 2 days and > 2 days of hospital admission, respectively.

BS = Blind subculture; BSI = bloodstream infection.

## RESULTS

### Blood culture results from July 2016 to October 2019.

**Figure 2.**
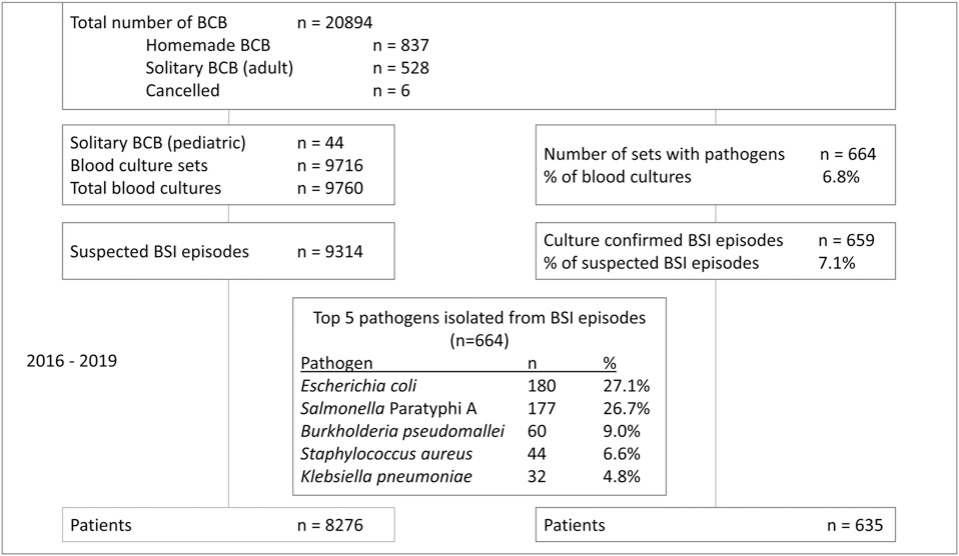
Breakdown of blood cultures sampled in Sihanouk Hospital Center of Hope from July 2016 to October 2019 (periods 2 and 3). * In five episodes, two different pathogens were retrieved from the same culture.

From July 2016 to October 2019, 20,894 bottles were sampled (Figure 2). After exclusion of homemade and solitary bottles, 19,523 bottles remained for analysis. These correspond to 9,760 cultures from 9,314 suspected episodes of BSI. They were obtained from 8,276 patients with median age of 52 years (2–98 years), including 247 children (age ≤ 18 years). Slightly more than half of patients were female (59.1%). Most of the suspected BSI episodes (96.5%) were community acquired (Table 2).

**Table 2:** Demographic and clinical data of patients with suspected and culture-confirmed BSI episodes from July 2016 to October 2019 (periods 2 and 3)

		All suspected BSI episodes	Non-confirmed BSI episodes	Culture-confirmed BSI episodes	*P*-value
No. of patients	–	8,276	7,641	635	
Female (% of number of patients)	–	4,892 (59.1)	4,539 (59.4)	353 (55.6)	0.13
Age, median (range) (years)	–	52 (2–98)	52 (2–95)	48.5 (9–98)	< 0.001
Total no. of BSI episodes	–	9,314	8,655	659	
Healthcare vs. community	Community- acquired	8,408 (95.7)	7,831 (95.8)	577 (93.5)	0.017
(% of number of episodes) (*n* = 8,790)*	Healthcare- associated	382 (4.3)	342 (4.2)	40 (6.5)	
Antibiotic treatment† (% of number of episodes) (*n* = 9,113)*	Recent	3,737 (40.1)	3,439 (40.6)	298 (45.2)	0.017
	None	5,376 (57.7)	5,027 (59.4)	349 (53.0)	

BSI = bloodstream infection. *P*-values were calculated for differences in parameters between confirmed and non-confirmed episodes, using a multivariate logistic regression model of (suspected) episodes during periods 2 and 3.

* Episodes for which no data were available were not included in this analysis.

† Recent antibiotic treatment was defined as administration of antibiotics in the last 2 weeks.

In 7.1% of suspected BSI episodes, a pathogen was recovered from blood culture. In total, 664 pathogens were recovered. There were five polymicrobial infections, that is, episodes for which two different pathogens were recovered. The most frequent pathogens were *Escherichia coli*, *Salmonella* Paratyphi A, and *B. pseudomallei* (Figure 2), which accounted together for 62.8% of all recovered pathogens. The contamination rate expressed per bottle and per culture was 2.7% and 5.1%, respectively. In 26 of 496 contaminated cultures, an identical contaminant was found in both bottles (5.2%).

Suspected episodes of patients under antibiotic treatment (antibiotic administered 2 weeks or less ago) showed growth more often (8.0%) than episodes of patients not under antibiotic treatment (6.5%) (*P* = 0.017; Table 2). Suspected healthcare-associated infections were also associated with higher growth rates (*P* = 0.017; Table 2). The mean blood volume sampled per bottle was 9.51 mL (SD 1.90); this was significantly higher than the mean volume of the period 2010–2015, when it was 7.99 mL (*P* < 0.0001). Correct filling rate (between 8 mL and 12 mL) increased from 59.5% of all bottles to 79.4% (*P* < 0.001).

Blind subculture yielded 184 pathogens, representing 27.7% of all recovered pathogens. All contaminants found on BS (*n* = 81) were recovered in the period July 2018 to October 2019, when the medium for BS was changed to blood agar: this allowed the growth of Gram-positive organisms. The most commonly isolated pathogen on BS was *Salmonella* Paratyphi A (*n* = 71; 38.6%), followed by *B. pseudomallei* (*n* = 40; 21.5%) and *E. coli* (*n* = 25; 13.6%). Proportionally, BS contributed most to detection of *B. pseudomallei*, yielding 66.7% of all recovered *B. pseudomallei*.

### Comparison of yield between the different surveillance periods.

Pathogen growth rate per suspected episode was 10.2% in period 1. In period 2, pathogen growth rate decreased to 7.1% (*P* < 0.001). In period 3, growth rate remained at 7.0%. The absolute number of cultures sampled per month increased during this period, from 177 per month to 239 per month in period 2 and to 252 cultures per month in period 3, as shown in Table 3. As also shown in this table, the recovery of pathogens in absolute numbers remained stable over the whole surveillance period (*P* = 0.17).

**Table 3:** Comparison of total blood culture yield and retrieval of *B. pseudomallei* and *Salmonella* Paratyphi A over different surveillance periods

	Period 1 (2010–2015)	Period 2 (2016–2018)	Period 3 (2018–2019)	Test for trend over time
Number of months of surveillance	66	24	16	–
Pathogen growth rate	10.7%	7.1%**	7.0%	*P* < 0.001
Total number of cultures sampled	11,671	5,732	4,028	–
Mean per month	177	239**	252**	*P* < 0.001
Total number of pathogens retrieved	1,087	387	277	–
Mean per month	16.5	16.1	17.3	*P* = 0.25
Retrieved on BS (% of all pathogens)	73 (6.7)	37 (9.6)	125 (45.1) **	*P* < 0.001
Total number of *B. pseudomallei* retrieved (% of all pathogens)	90 (8.3)	48 (12.4)**	12 (4.3)**	–
Mean per month	1.4	2.0*	0.8**	*P* = 0.53
Retrieved on BS (% of all *B. pseudomallei*)	55 (55.6)	28 (56.0)	9 (75.0)	*P* = 0.002
Total number of *Salmonella* Paratyphi A (% of all pathogens)	147 (13.5)	74 (19.1)	110 (39.7)	–
Mean per month	2.2	3.1*	6.9**	*P* < 0.001
Retrieved on BS (% of all *Salmonella* Paratyphi A)	1 (0.1)	0 (0.0)	71 (65.5)**	*P* < 0.001

BS = blind subculture; *B. pseudomallei = Burkholderia pseudomallei*. Pathogen growth rate was defined as proportion of blood cultures showing growth of a pathogen. Statistically significant differences with the previous surveillance period are indicated with asterisks (* = *P* < 0.05; ** = *P* < 0.001). Logistic and Poisson regressions were used to detect a linear trend over time. This table shows that pathogen growth rate significantly declined in periods 2 and 3, but that the total number of pathogens retrieved remained stable. The total number of *Salmonella* Paratyphi A retrieved showed an increasing trend, whereas the total number of *B. pseudomallei* retrieved increased in period 2 but decreased significantly in period 3.

Table 4 shows the output of the multivariate and segmented regression models for the different surveillance periods. Pathogen growth rate per culture was stable over time within each surveillance period, but a significant decline in the growth rate was seen after the first intervention (July 2016). Prior antibiotic treatment had a negative effect on growth rates in period 1, but a positive, albeit not statistically significant, effect in periods 2 and 3. Contrary to expectations, higher sampled volume was not associated with better recovery of pathogens, nor was increased incubation delay associated with worse recovery of pathogens.

**Table 4:** Effect of age, gender, hospitalization status, prior antibiotic use, volume sampled, incubation delay, and time (by quarter) on pathogen growth rate, within the different surveillance periods

Variables	OR period 1 (95% CI)	OR period 2 (95% CI)	OR period 3 (95% CI)
Age (years)	1.00 (1.00–1.00)	0.99 (0.99–1.00)	0.99 (0.98–0.99)**
Gender (male vs. female)	0.99 (0.87–1.14)	1.40 (1.09–1.80)*	0.93 (0.72–1. 20)
Hospitalized (yes vs. no)	0.99 (0.81–1.21)	1.15 (0.81–1.64)	1.53 (1.01–2.32)*
Antibiotics (prior antibiotics vs. no prior antibiotics)	0.78 (0.67–0.90)**	1.17 (0.83–1.65)	1.13 (0.87–1.47)
Time since start surveillance (quarter)	1.00 (0.92–1.30)	1.03 (0.97–1.10)	1.06 (0.97–1.14)
Cultured volume (mL)	0.98 (0.96–1.00)	1.01 (0.97–1.05)	0.98 (0.94–1.02)
Incubation delay (day)	1.09 (0.92–1.30)	0.80 (0.37–1.72)	0.85 (0.58–1.25)
Change in pathogen growth rate after each intervention (assuming two breakpoints)	–	0.71 (0.48–1.05)	0.69 (0.45–1.04)
Change in pathogen growth rate after the first intervention (assuming one breakpoint)	–	0.74 (0.56–0.99)*	–

OR = odds ratio. The magnitude of the effect is expressed as an OR of growth with 95% CI resulting from a multivariate logistic regression model for the three different surveillance periods. The effect of the laboratory interventions on growth rate was modeled with a segmented regression using either one (July 2016) or two “breakpoints” (July 2016 and July 2018). Odds ratios statistically different from 1 are indicated with asterisks (* = *P* < 0.05; ** = *P* < 0.001). Pathogen growth rate does not change over time within the surveillance periods, but baseline growth rate decreased with 26% after the first intervention (from period 1 to period 2).

Another important observation was the declining recovery of *B. pseudomallei* in period 3, when the BS was advanced to day 1 (Table 3). This followed a relative increase during period 2, when proportion rose from 8.3% to 12.4% (*P* = 0.02). The proportion of *B. pseudomallei* among all culture-confirmed BSI went down from 12.4% in period 2 to 4.3% (*P* < 0.001). Simultaneously, the proportion of *Salmonella* Paratyphi A further increased to 39.7% of all pathogens, demonstrating an obvious increasing trend since 2010 (Table 3).

#### Comparison of time to detection and time to colonies between the different surveillance periods.

The yield of BS increased significantly when the subculture was advanced; it rose from 6.7% of all retrieved pathogens to 9.6% by advancing the subculture to day 2; it further increased to 45.1% by advancing subculture to day 1 (Table 3).

**Figure 3.**
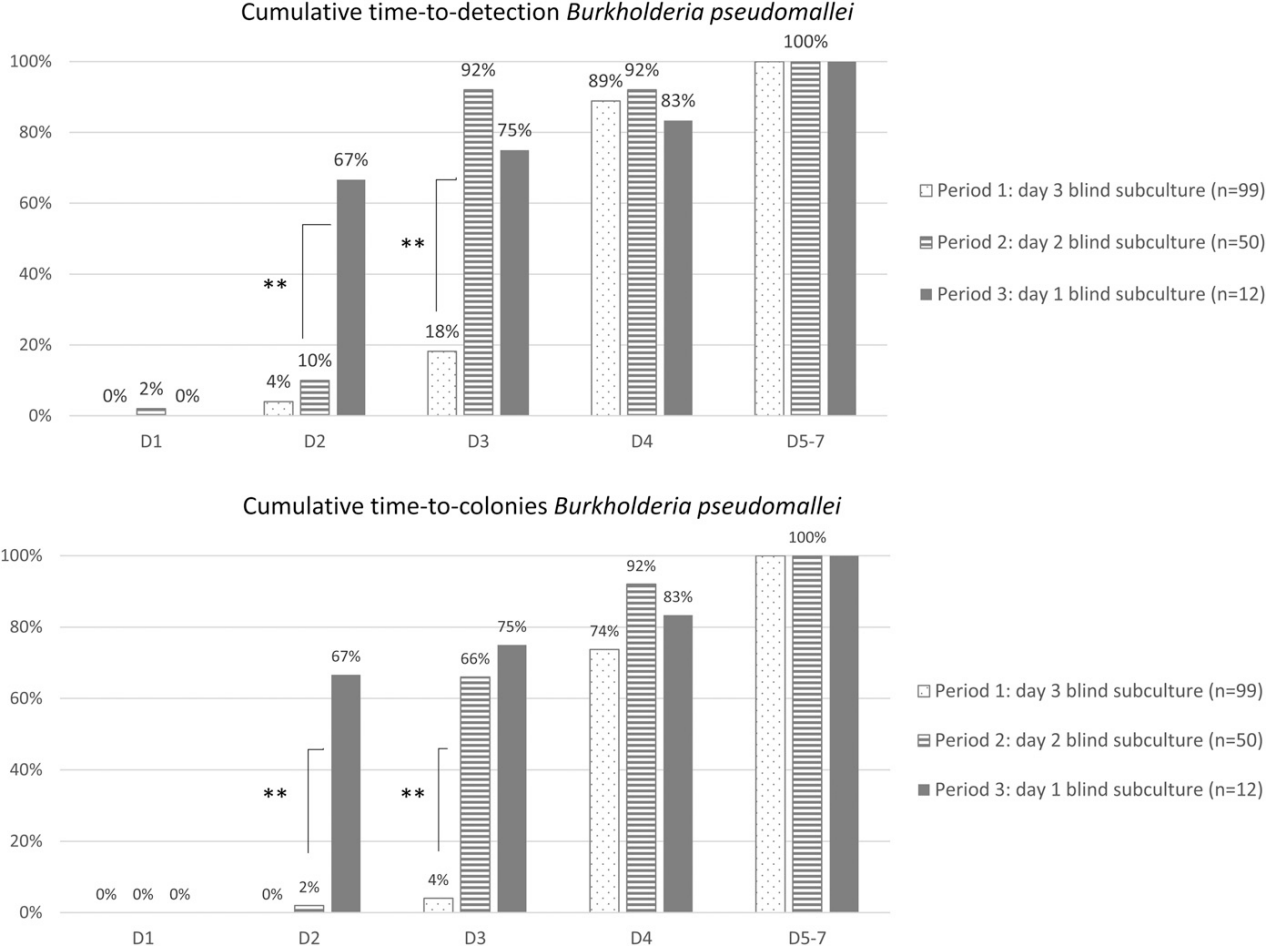
Cumulative time to detection (first sign of growth) and time to colonies for all pathogens except *Burkholderia pseudomallei*, comparing the three periods of surveillance. Statistically significant differences between periods of surveillance are indicated with asterisks (* = *P* < 0.05; ** = *P* < 0.001). This figure appears in color at www.ajtmh.org.

**Figure 4.**
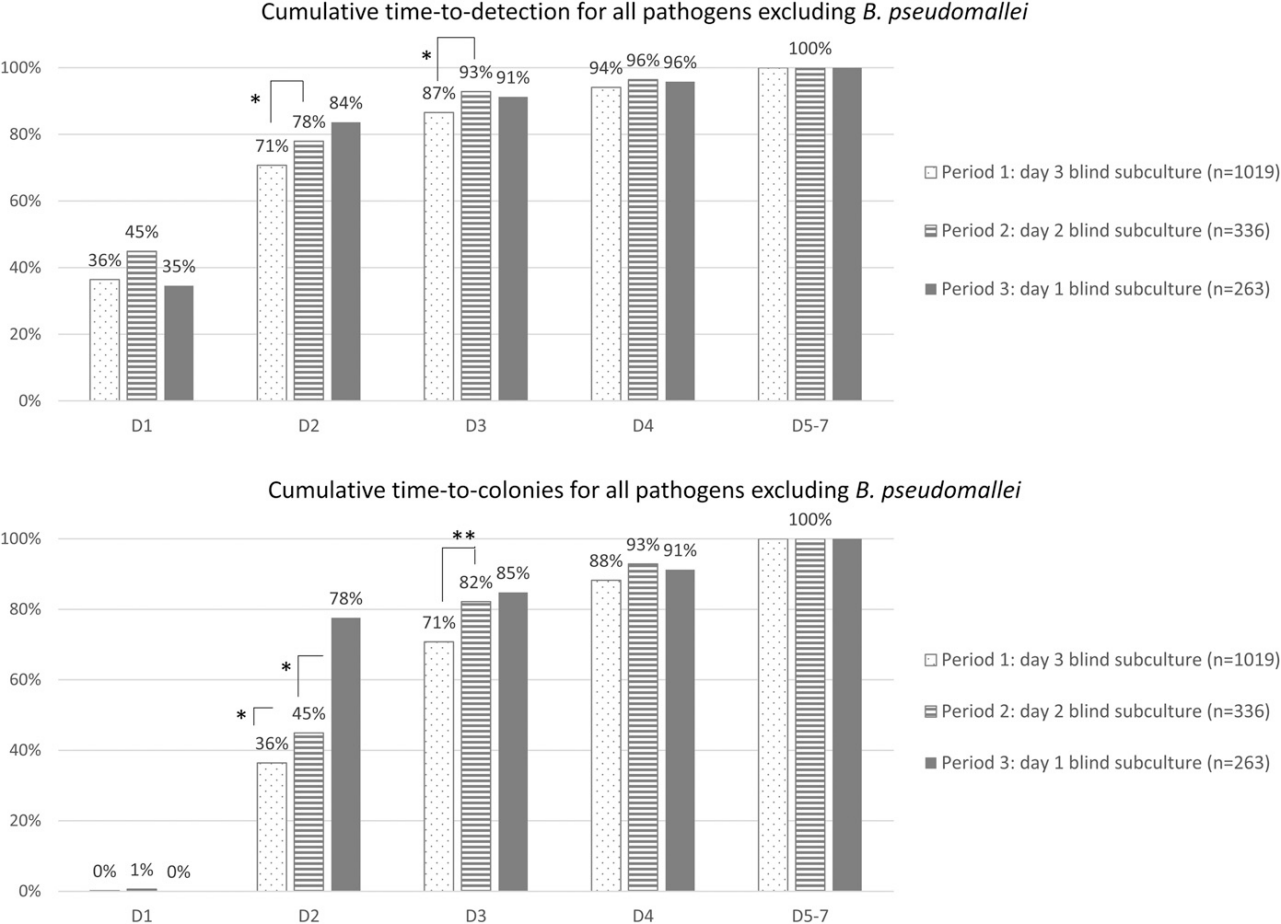
Cumulative time to detection (first sign of growth) and time to colonies for *Burkholderia pseudomallei*, comparing the three periods of surveillance. Statistically significant differences between periods of surveillance are indicated with asterisks (* = *P* < 0.05; ** = *P* < 0.001). This figure appears in color at www.ajtmh.org.

By advancing the BS to day 2 of incubation, the time to detection of growth in general, and specifically of *B. pseudomallei*, shortened significantly (Figures 3 and 4). The cumulative proportion of all pathogens (excluding *B. pseudomallei*) retrieved by day 3 (the day following the BS) increased from 86.6% to 92.9% (*P* = 0.002). For *B. pseudomallei*, cumulative proportion retrieved by day 3 increased rather spectacularly from 18.2% to 92% (*P* < 0.001).

Blind subculture on day 1 further shortened the time to detection. The proportion of total pathogens retrieved by day 2 increased from 69.0% to 82.9% (*P* < 0.001). Time to detection shortened for *B. pseudomallei*; cumulative growth on day 2 increased to 66.7% for all *B. pseudomallei* cultures, from only 10.0% when day 2 BS was performed (*P* < 0.001)(Figure 3).

Figures 3 and 4 also show the cumulative time to colonies; this is the interval between the moment of incubation to the moment colonies are available for identification and antibiotic susceptibility testing. A shorter time to colonies therefore leads to faster actionable results for the clinician. The impact of advancing the BS on time to colonies is even greater than that of advancing the BS on time to detection. A day 2 BS led to 80.1% of all pathogens (including *B. pseudomallei*) showing colony growth by day 3, versus 67.2% with a day 3 BS (*P* < 0.001). Similarly, advancing the subculture to day 1 led to a recovery of colonies on day 2 of 77.1%, versus only 39.3% when a day 2 BS was performed (*P* < 0.001).

Even for pathogens that do not benefit from BS in terms of time to detection, BS may lead to faster recovery of colonies. For example, advancing the BS to day 1 did not significantly shorten the time to detection for *S. aureus* (cumulative detection by day 2 actually slightly decreased from 53.8% in period 2–50.0% in period 3), but it did significantly shorten the time to colonies: when performing BS on day 1, colonies were available for 44.4% of all grown *S. aureus* cultures by day 2, versus only 15.4% when BS was performed on day 2 (*P* = 0.04).

## DISCUSSION

This analysis of blood culture data from SHCH studied the effect of implementation of procedural changes in the laboratory on important quality indicators such as time to detection of pathogens and blood culture yield.

Advancing the BS to day 2 led to shorter time to detection overall and higher relative recovery of *B. pseudomallei*. This higher recovery may also have been caused by the change of an aerobic–anaerobic pair to an aerobic–aerobic pair; a previous analysis had shown that *B. pseudomallei* grew mainly in the aerobic bottle of a blood culture set (70/99 *B. pseudomallei* cultures grew only in the aerobic bottle).^8^ When an aerobic–aerobic pair was used, 10 of 62 cultures growing *B. pseudomallei* showed growth in just one bottle (16.1%). Had an aerobic–anaerobic pair been used, half of these infections would theoretically have been missed. However, *B. pseudomallei* incidence may also have increased in the region during that period; epidemiological data on *B. pseudomallei* are scarce, but Calmette Hospital reported their incidence of *B. pseudomallei* to the Melioidosis Research Coordination Network, and observed a steady increase in identified cases from 2013 to 2018.^14^

The overall pathogen growth rate per suspected episode decreased from 10.2% in period 1 to 7.1% for periods 2 and 3. This is still within the recommendations of blood culture guidelines, which recommend a pathogen growth rate between 6% and 12%,^15^ but the decrease was nonetheless significant and a cause of concern. Reasons for a declining growth rate can be related to efficiency of recovery of pathogens or to sampling indications. The indications for blood culture sampling have not changed between 2010 and the present, but we observed a sharp increase in the number of blood cultures sampled when comparing periods 2 and 3 with period 1 (Table 3). Because of hospital budget problems, the number of patients was limited in the period before 2016. The hospital went to full function of both outpatient departments and hospital beds since 2016. The number of pathogens detected per month, however, remained stable. These results suggest the drop in pathogen growth rate may have been caused by increased sampling, rather than suboptimal recovery of pathogens.

The replacement of an anaerobic bottle by another aerobic bottle led to an expected decrease in recovery of strictly anaerobic pathogens; it is however unlikely this would have had a significant effect on pathogen growth rate, as only 34 strictly anaerobic pathogens were recovered in period 1 (3.0% of all grown cultures; 0.3% of all sampled blood cultures). The omission of anaerobic bottles is contested, as many authors point out that anaerobic infections may not always be correctly predicted by clinical signs; antibiotic resistance among anaerobic bacteria is increasing, and some facultative pathogens show faster growth in anaerobic than aerobic bottles.^16–19^ However, our own evaluation of the use of anaerobic bottles in SHCH showed slower and less efficient overall pathogen growth in anaerobic bottles.^8^ Given the importance of the obligate aerobic pathogen *B. pseudomallei* in this setting and the inability of the laboratory to perform anaerobic antibiotic susceptibility testing due to technical limitations, we believe anaerobic bottles are of limited value in SHCH, which is in line with findings of other studies.^20–22^

Further advancing the BS to day 1 increased the overall yield of BS and detection of *B. pseudomallei* by day 2 of incubation significantly (Table 3, Figure 3). The effect of the advanced BS on time to detection for pathogens other than *B. pseudomallei* was not statistically significant (Figure 4). Overall pathogen growth rate remained stable (despite further increase in numbers of cultures sampled as shown in Table 3), but relative frequency of *B. pseudomalle*i decreased markedly. This relative decrease may have been partly due to a resurgence of the previously described *Salmonella* Paratyphi A outbreak in Cambodia that was observed simultaneously (Table 3).^23^ However, the absolute number of *B. pseudomallei* retrieved per month decreased significantly as well. Unfortunately, no official data about epidemiology of *B. pseudomallei* in this region are available after 2018, but personal communication from the Diagnostic Microbiology Development Program, working in Takeo Hospital, did not reveal a similar decrease in *B. pseudomallei* incidence in Takeo Province. Given the importance of BS in the detection of *B. pseudomallei*, its overall slow growth, as described previously,^8^ and scarce visual signs of growth of non-fermenting Gram-negative organisms, it is possible that growth of *B. pseudomallei* was missed by advancing the subculture.

Optimal timing of BS largely depends on the goal of BS. An early BS (within 12–24 hours of incubation) decreases the time to detection,^24–26^ whereas a late BS serves as a final check for growth, to ensure no pathogens are missed.^10^ The “Clinical Microbiology Procedures Handbook” recommends a late subculture after 72 hours of incubation,^10^ whereas Clinical & Laboratory Standards Institute recommends performing subculture after 24–48 hours of incubation,^27^ and Cumitech advises an ever earlier subculture after 12–18 hours of incubation.^15^ A study looking at the optimal timing for subculture specifically for the detection of *B. pseudomallei* found that an early day 1 subculture confirmed growth of 52.3% of all *B. pseudomallei* cultures.^28^ An additional BS on day 2 yielded an extra 28.5% of grown cultures.^28^ However, the results of our study suggest that performing only a day 1 BS may decrease the overall recovery of *B. pseudomallei*. For settings where *B. pseudomallei* is an important pathogen, we therefore recommend BS on day 2 of incubation as the best trade-off between time to detection and pathogen growth rate.

The potential benefits of early BS are not limited to earlier detection of pathogens; it also leads to earlier recovery of colonies, which can be immediately used for supplementary testing. Fast results of antibiotic susceptibility testing, for example, have a large impact on patient management. These tests depend on colony growth; therefore, shorter time to colonies leads directly to faster actionable results, clinical decision-making, and directed treatment. This is particularly important in a context of increased multidrug-resistant pathogens and endemic *B. pseudomallei* infection, as empirical treatment may not adequately cover these pathogens.^29–31^ As shown in Figures 3 and 4, the effect of BS was more pronounced on time to colonies than on time to detection of pathogens.

Performing a BS on all bottles adds a substantial workload, and thus costs to a blood culture system. The replacement of an anaerobic by an aerobic bottle doubled the workload for BS, as this practice is only recommended for aerobic bottles.^15^ Pathogen growth rates of blood cultures are low (recommended pathogen growth rate is 6–12%, implying the large majority of sampled cultures do not show growth)^15^; therefore, most of the work associated with performing BSs does not result in faster recovery of pathogens. Faster pathogen detection must therefore be balanced against increased labor costs. Labor costs are generally lower in most low-resource than in high-resource settings; therefore, BS is probably more efficient in these settings. The added value of increased and faster recovery of pathogens with BS should be balanced against this increased workload and an increased risk of contamination of the blood culture bottles.

The change in blood culture bottles (FA FAN to FA Plus Bact/ALERT bottles) could have contributed to faster growth, although there was no significant change in visual signs of positivity on day 1 (the only day not affected by changes in BS timing); cumulative first growth on day 1 increased only slightly from 33.1% to 36.7% after implementation of the new bottles (*P* = 0.13). However, cumulative time to detection by day 2 increased significantly for pathogens not including *B. pseudomallei* in period 2, indicating a possible bottle effect as BS could not have impacted growth on day 2 yet (Figure 4). Moreover, the presence of resins in the FA Plus blood culture medium may have had an impact on growth rates of blood cultures taken under antimicrobial therapy, as resins have been shown to successfully inhibit the effect of antibiotics present in serum.^32–34^ This is indeed suggested by the fact that prior antibiotic therapy had a negative effect on the growth rate in period 1 (odds ratio [OR] of 0.78, *P* < 0.001), whereas cultures under antibiotic therapy were more likely to show growth in periods 2 and 3, after the implementation of the resin-containing bottles (OR: 1.17 and 1.13, respectively, *P* > 0.059) (Table 4).

Another effect of the change in bottles could be related to visual detection of growth. The bottles are currently used off-label, as they are specifically constructed for use in an automated incubator. Other studies have used a similar approach, but also with bottles not containing resins.^35^ Visual growth characteristics such as turbidity and puff balls can be less clearly visible in resin-containing bottles.^36^ The observed decrease in pathogen growth rate could be related to less efficient detection of growth, especially of organisms that cause little visible change in the color indicator.

As compared with surveillance period 1, volume of blood culture bottles was higher, and significantly more bottles were correctly filled in periods 2 and 3. A monthly feedback system was implemented in 2014, whereby quality indicators such as bottle weight and contamination rate were presented to nursing teams, creating increased awareness of correct blood culture sampling procedures. The success of this feedback system is further highlighted by the observation that these filling volumes compare favorably with those reported from high-income countries, despite the limitations inherent to low-resource settings.^37,38^ Volume did however not appear to have an impact on growth rates. This could be explained by the assumption that sampling high volumes of blood from severely ill patients is more difficult and less easily obtained; these patients are however more likely to suffer from BSI. No information on disease severity was available for this study.

Even with early BS, time to detection of many pathogens, most notably non-fermenters such as *B. pseudomallei*, remains behind that of automated systems.^9,39–41^ A possible explanation for this observation is faster detection of growth by the algorithms in the automate software than visual assessment of the indicator. However, improved time to detection may also be related to faster growth. Blood culture automates agitate the blood cultures continuously during incubation, which has been shown to increase recovery of pathogens, probably due to increased oxygenation.^42–46^ Moreover, temperature in automated incubators is presumably more stable than that in a conventional incubator, which has to be opened in its entirety each time a bottle is added or removed.

The limitations of this study are its observational nature and the simultaneous implementation of multiple laboratory changes. Causal effects of one factor on outcomes such as yield and speed of detection are therefore hard to pinpoint. Moreover, other changes in hospital policy and in underlying population trends could have influenced our results. In the case of time to detection and time to colonies, however, there is strong evidence that advancing the BS led to faster recovery of pathogens. The shifting proportions of different key pathogens could have been reflections of changing underlying epidemiology, although there are indications that the laboratory changes affected detection of *B. pseudomallei*. Another limitation was the lack of information on time delays (in hours) between the sampling of blood cultures and the time of incubation, which could have provided an alternative hypothesis for poor detection rates of *B. pseudomallei*. We found no relation between incubation delay and growth; however, delay was only measured in days, precluding demonstration of the effect of more subtle time delays. It has been shown that non-fermenting Gram-negative organisms can go into a bacteriostatic phase when pre-incubated, inhibiting their detection in automated systems.^47^ However, we believe this problem is less important in this setting, as no automated detection was used, and pre-incubation is unlikely to impact yield of BS or visual detection. Moreover, the lack of terminal subculture (additional subculture at the end of the incubation period) during this study precludes conclusions on how many pathogens were missed by advancing the BS. It was therefore decided to temporarily introduce a terminal subculture in SHCH to revalidate the BS procedure.

In conclusion, this study demonstrates that rigorous follow-up of blood cultures and quality indicators is possible in low-resource settings and can lead to significant improvements in laboratory and sampling procedures. A faster detection of important pathogens is possible by relatively inexpensive methods such as introducing or advancing a BS early in the period of incubation. This study also demonstrates the possible risks associated to such changes, and the need for continuous monitoring. Based on these study results, it was decided that BS on day 2 was the preferred subculture timing for SHCH, optimizing the balance between time to detection and pathogen recovery. Despite the best efforts, time to detection of these manual blood cultures systems still lags behind that of automated blood culture systems. Robust, affordable detection systems to further shorten time to detection for low-resource settings are warranted.
